# *Andrographis paniculata* (Burm. f.) Wall. ex Nees: A Review of Ethnobotany, Phytochemistry, and Pharmacology

**DOI:** 10.1155/2014/274905

**Published:** 2014-12-24

**Authors:** Md. Sanower Hossain, Zannat Urbi, Abubakar Sule, K. M. Hafizur Rahman

**Affiliations:** ^1^Department of Biotechnology, Kulliyyah of Science, International Islamic University Malaysia, 25200 Kuantan, Pahang, Malaysia; ^2^Department of Microbiology, School of Applied Sciences, Federal Polytechnic Institute, Nasarawa, Nigeria; ^3^Department of Basic Medical Sciences, Kulliyyah of Pharmacy, International Islamic University Malaysia, 25200 Kuantan, Pahang, Malaysia

## Abstract

As aboriginal sources of medications, medicinal plants are used from the ancient times. *Andrographis paniculata* is one of the highly used potential medicinal plants in the world. This plant is traditionally used for the treatment of common cold, diarrhoea, fever due to several infective cause, jaundice, as a health tonic for the liver and cardiovascular health, and as an antioxidant. It is also used to improve sexual dysfunctions and serve as a contraceptive. All parts of this plant are used to extract the active phytochemicals, but the compositions of phytoconstituents widely differ from one part to another and with place, season, and time of harvest. Our extensive data mining of the phytoconstituents revealed more than 55 *ent*-labdane diterpenoids, 30 flavonoids, 8 quinic acids, 4 xanthones, and 5 rare noriridoids. In this review, we selected only those compounds that pharmacology has already reported. Finally we focused on around 46 compounds for further discussion. We also discussed ethnobotany of this plant briefly. Recommendations addressing extraction process, tissue culture, and adventitious rooting techniques and propagation under abiotic stress conditions for improvement of phytoconstituents are discussed concisely in this paper. Further study areas on pharmacology are also proposed where needed.

## 1. Introduction

Medicinal plant is an integral part of human life to combat the sufferings from the dawn of civilization [1]. It is estimated that more than 80,000 of total plant species have been identified and used as medicinal plants around the world [2]. Among these plants, more than 1300 plant species have been used traditionally in Malaysia where the knowledge is being passed down from generation to generation [3]. The indigenous medicinal plants and plant-derived drugs are the potential source of alternative medicine and are extensively used to treat various health ailments [4]. Use of the medicinal plants is a core component at primary health care level due to availability, acceptability, compatibility, and affordability. Dependency on these medicinal plants varies from country to country. It is estimated that about 75–80% of people of developing countries and about 25% of people of developed countries depend either directly or indirectly on medicinal plants for the first line of treatment [3, 5]. Therefore, people are encouraging indigenous production and processing of these medicinal plants to use in different cultures and religion for the treatment of various diseases. Moreover, importance and uses of medicinal plants are also stated in different religious books (i.e., the Holy Qur'an, the Bible). About 19 medicinal plants and 176 medicinal plants are mentioned in the Holy Qur'an [6] and the Holy Bible [7], respectively.

*Andrographis paniculata* (Burm. f.) Wall. ex Nees (AP) is an important medicinal plant and widely used around the world. It belongs to the family Acanthaceae. AP is used as a traditional herbal medicine in Bangladesh, China, Hong Kong, India, Pakistan, Philippines, Malaysia, Indonesia, and Thailand [8, 9] and is ethnobotanically used for the treatment of snake bite, bug bite, diabetes, dysentery, fever, and malaria [3]. In the Unani and Ayurvedic medicines, AP is one of the mostly used medicinal plants [8]. In recent times, commercial preparations of this plant extracts are also used in certain countries. However, the preparations yet need to be standardized for their better efficacy. The aerial part of AP is most commonly used; its extracts contain diterpenoids, diterpene glycosides, lactones, flavonoids, and flavonoid glycosides. Whole plant leaves and roots are also used as a folklore remedy for different diseases in Asia and Europe [8, 10]. AP has been reported to have a broad range of pharmacological effects including anticancer [11–18], antidiarrheal [19, 20], antihepatitis [21, 22], anti-HIV [23], antihyperglycemic [24–27], anti-inflammatory [28–32], antimicrobial, antimalarial [33, 34], antioxidant [35–37], cardiovascular [38, 39], cytotoxic [23], hepatoprotective [40–52], immunostimulatory [53–57], and sexual dysfunctions [58].

Since the AP is used for the treatment of many diseases in traditional medicinal systems, its intended benefits need to be evaluated critically. Therefore, this paper reviews the ethnobotany, some agronomic techniques, isolation and characterization of phytoconstituents, and pharmacological properties of AP. Additionally, chemical properties, biological functions, and possible mode of actions of phytoconstituents are also entertained. The literature searches were conducted in worldwide accepted scientific database PubMed (http://www.ncbi.nlm.nih.gov/pubmed), ScienceDirect (http://www.sciencedirect.com/), Scopus (http://www.scopus.com/), Web of Science (http://webofknowledge.com/), Springer Link (http://link.springer.com/), Wiley Online Library (http://onlinelibrary.wiley.com/), and advance search in Google scholar (http://scholar.google.com.my/), as well as recognized books, abstract, and some nonimpact, and nonindexed journals. The search strings used were “*Andrographis paniculata*” or “King of Bitters” or “Kalmegh” or “Hempedu bumi” or “Andrographolide.” Further relevant papers were searched in the aforementioned databases from the list of references of all available papers. The authors went through more than 300 full papers and a total of 243 peer-reviewed papers focused on plant description, agronomic techniques, traditional uses, bioactive compound extraction, phytoconstituents, and pharmacology properties (such as common cold, anti-inflammatory, antihyperglycemic, hepatoprotective, antibacterial, antiviral, antiparasitic, anticancer, immunomodulatory, cardiovascular, antihyperlipidemic, sexual dysfunctions, contraceptive, safety, and toxicity effects) were selected for this review. We briefly discussed recent scientific findings and proposed some fields where further study is needed.

## 2. Botany

### 2.1. Origin and Distribution

*A. paniculata* is native to Taiwan, Mainland China, and India. It is also commonly found in the tropical and subtropical Asia, Southeast Asia, and some other countries including Cambodia, Caribbean islands, Indonesia, Laos, Malaysia, Myanmar, Sri Lanka, Thailand, and Vietnam [59–61]. This plant is also found in different phytogeographical and edaphic zones of China, America, West Indies, and Christmas Island [61].

### 2.2. Plant Description

*A. paniculata* is an important medicinal plant of* Andrographis* genus. A total number of species of this genus varied in different reports, which comprises either 19 [64, 65], 28 [59, 66], 40 [62, 67], or 44 [68] species. The exact numbers of species of* Andrographis* genus are not validated yet. Total number of chromosomes of AP is 25 and 50 in gametophytic [69] and sporophytic [70] count, respectively. In addition, genotypic differences are important considerations to find out high yielding germplasms.

**Figure 1:**
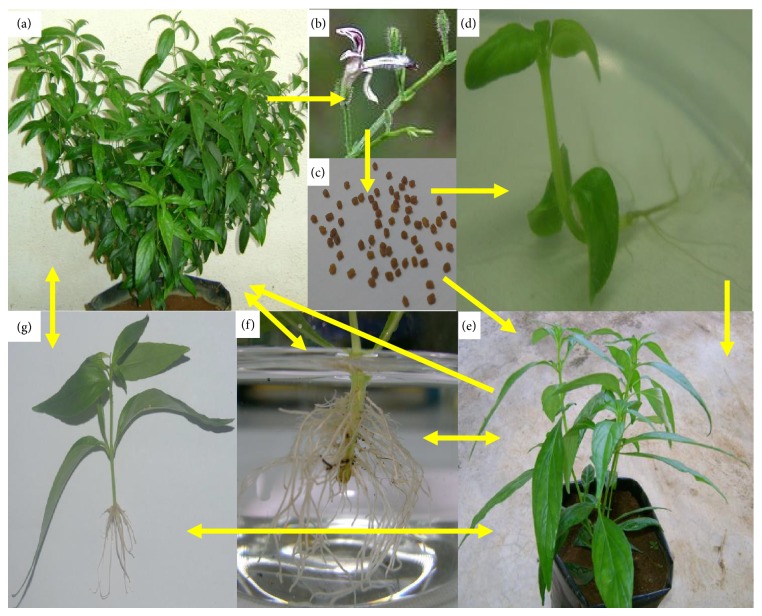
Morphology of* Andrographis paniculata*. (a) Mature* A. paniculata *in polybag stage, (b) flowering stage, (c) harvested seeds, (d)* in vitro* seedling, (e) young* A. paniculata* in polybag, (f) adventitious roots of* A. paniculata*, and (g) vegetative seedlings. Single direction of arrow indicates the developmental stages and both directions of arrow denote vegetative propagation of plant (Photographs are taken from M.S. Hossain's research work, except (b)).

*A. paniculata* is an annual, branched, erect, and herbaceous plant which grows in hedgerows throughout the plane lands, hill slopes, waste ground, farms, moist habitat, seashores, and roadsides. It also can be cultivated in garden. Moist shady places, forests, and wastelands are preferable for their well development [59, 71]. The morphological and physiological data of AP are presented in Table 1 and Figure 1. This plant grows abundantly in Southern and Southeastern Asia including India, Java, Sri Lanka, Pakistan, and Indonesia, while it is cultivated in India, China, Thailand, Brunei, Indonesia, the West Indies such as Jamaica, Barbados, and Bahamas, Hong Kong, and the tropical areas in America and also in southwestern Nigeria [10, 71].

### 2.3. Taxonomic Hierarchy

Taxonomic hierarchy is as follows:

 Domain: Eukaryota,
 Kingdom: Plantae,
 Subkingdom: Tracheobionta,
 Superdivision: Spermatophyta,
 Division: Angiosperma,
 Class: Dicotyledonae,
 Subclass: Gamopetalae,
 Series: Bicarpellatae,
 Order: Personales,
 Family: Acanthaceae,
 Subfamily: Acanthoideae,
 Tribe: Justiciae,
 Subtribe: Andrographideae,
 Genus:* Andrographis,*
 Species:* A. paniculata* (Burm. f.) Nees [71, 73, 74].

### 2.4. Vernacular Names

Generally, the tree is known as “King of Bitters” for its extremely bitter taste. In Malaysia, AP is traditionally known as “hempedu bumi” (bile of the earth). This plant has different names in different languages. The vernacular names of AP are presented in Table 2. In addition, local people easily recognize plant species with vernacular names instead of binomial names.

### 2.5. Agronomic Techniques

Plants conventionally grow via seed culture. Planting and harvesting time has influenced the yield of plant [153, 154]. Usually, May to July is the recommended time for sowing AP seeds with a spacing of 30 × 15 cm that resulted in a plant density of 222 thousands plants/ha and average biomass of 3 t/ha [155, 156]. However, seed dormancy is a major constrain in AP cultivation commercially [157, 158]. Although hormonal media and hot water treatment have been suggested to overcome this problem [159, 160], this technique is not enough to meet the commercial quantities required due to variability among the seed-derived progenies and scanty and delayed rooting of seedlings [161, 162]. Therefore, nonconventional propagation methods such as plant tissue culture techniques are alternative methods to produce plenty of plantlets within a short time and improve phytochemical contents in AP. Tissue culture techniques have been applied for large scale propagation of AP [162–164]. Dandin and Murthy [164] reported that* in vitro* regenerated AP contains higher amount of andrographolide compared to the mother plants. Optimum production of andrographolide can be achieved in a short span by employing the suspension cultures of AP [165]. Plant tissue culture has also been successfully used to form new flavones from callus culture [166–168].

## 3. Ethnobotanical Uses

Ethnobotanically, the leaves and roots of AP have been used since centuries in Asia and Europe to cure the wide spectrum of health ailments. However, the whole plant is also used for certain limited purposes. Due to its “cold property” activity, it is recommended to be used to get rid of the body heat in fevers and to dispel toxins from the body. The plants are also recommended for the use in cases of leprosy, gonorrhea, scabies, boils, skin eruptions, and chronic and seasonal fever for its high “blood purifying” properties [8, 169]. The overall traditional uses of AP in different traditional medicinal systems (TMS) or countries are pointed out in Table 3. In addition, it is also widely used for medicinal purposes by the traditional practitioners, tribes, or community as a folklore remedies in different countries [9].

## 4. Phytoconstituents

**Figure 2:**
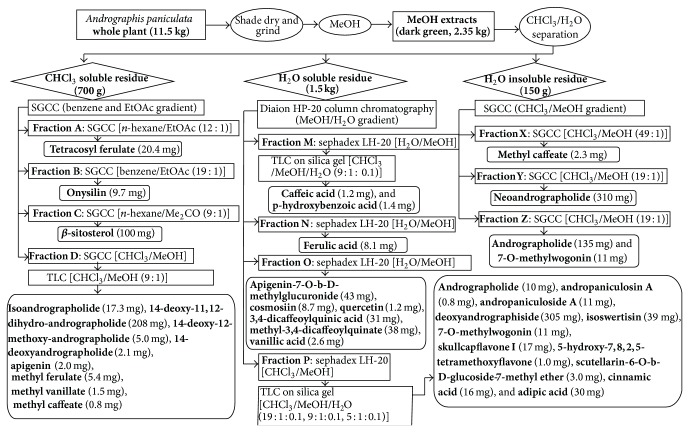
Extraction of pure compounds of* A. paniculata* from MeOH extracts. Yield of pure compounds is in bracket in mg. Their structures have been elucidated mainly by 1D and 2D NMR and MS spectroscopic methods. SGCC: silica gel column chromatography; TLC: thin layer chromatography, MeOH: methanol; EtOAc: ethyl acetate, CHCl_3_: chloroform (adopted from [72]).

The aerial parts of AP have been described for its innumerous use in the extraction of phytoconstituents; however, leaves, stems, roots, and whole plants have also been reported for phytochemicals with pharmacological activities. The compositions of phytochemicals widely differ in terms of the part used, geography, season, and time of harvesting. M. Sharma and R. Sharma [76] reported that the highest amount of andrographolide, a major bioactive compound of AP, was found in the sample harvested after 110 days of cultivation followed by that just before flowering stage (130 days). The bioactive compounds were extracted with different types of solvents such as methanol (MeOH), ethanol (EtOH), hexane, acetone, acetone-water, chloroform (CHCl_3_), and dichloromethane from the whole plant, leaves, aerial parts, stems, and roots. Extraction procedure of bioactive compounds of AP from MeOH extracts, for example, is shown in Figure 2. In this extraction procedure, whole plant material of AP (11.5 kg) was shade dried, ground, extracted with methanol (10 L × 6) under reflux for 8 h, and filtered to give residue. After filtration, three residues of the extract were found: (i) CHCl_3_ residue (700 g), (ii) H_2_O soluble (1.5 kg), and (iii) H_2_O insoluble residue (150 g). The residues were then chromatographed to get specific fractions. These fractions were further chromatographed and followed several procedures to identify specific compound (Figure 2). A total of 32 bioactive compounds with seven* ent-*labdane diterpenoids, twelve flavonoids, and two quinic acid derivatives have been isolated and characterized by this procedure. Previous phytochemical studies of AP have reported more than 55* ent*-labdane diterpenoids, 30 flavonoids, 8 quinic acids, 4 xanthones [132, 150], and 5 noriridoids, namely, andrographidoids A, B, C, D, and E [170, 171].

Zhang et al. [172] reported 3 new* ent*-labdane diterpenoids, namely, 19-norandrographolides A, B, and C from the ethanol extracts of the aerial parts of AP. Their structures have been established by HRESIMS and NMR spectral data in combination with X-ray crystallographic analysis; thus the 19-norandrographolide-A was identified as 3-dehydro-14-deoxy-19-norandrographolide. However, there is no report for pharmacological activity of these three compounds. The quinic acids were extracted only from the whole plants using the methanol solvents and they are functioning as an antiplatelet aggregator [72]. Recently, Arifullah et al. [141] demonstrated moderately potent antimicrobial and antioxidant activity of andrographolide and echiodinin extracted from acetone and methanol extracts of* in vitro* leaf callus of AP. Their structure elucidation was determined by electrospray ionization, MSD, NMR, and IR spectra. The yield of this compound was higher compared to the field plant. On the other hand, a novel flavonoid, 7, 8-dimethoxy-2′-hydroxy-5-O-*β*-d-glucopyranosyloxyflavone, together with 15 known flavonoids was isolated from the aerial parts of AP. The structure was elucidated on the basis of chemical and spectroscopic analysis. Significant antiproliferative activity of these flavonoids against human leukaemia HL-60 cell was also investigated [142]. Dua et al. [143] investigated four xanthones (1,2-dihydroxy-6,8-dimethoxyxanthone; 1,8-dihydroxy-3,7-dimethoxyxanthone; 3,7,8-trimethoxy-1-hydroxyxanthone; 4,8-dihydroxy-2,7-dimethoxyxanthone) from roots of AP using CHCl_3_ fraction and purity confirmed by HPLC. Xu et al. [171] isolated and established structure of 5 rare types of noriridoids with a known iridoid curvifloruside F from the ethanol extracts of roots. They also assayed antibacterial activity of these compounds, but none showed any inhibitory activity (MIC > 100 *μ*g/mL). In this review, we provided available information on around 46 isolated phytoconstituents of AP with structure, chemical properties, IUPAC name, reported pharmacological activities, mode of extraction, and parts used (Table 4).

## 5. Potential Pharmacology

Extensive use of AP in traditional medicinal system has proven its efficacy over the past three decades. Several researches including* in vitro*,* in vivo* (animal), and clinical (human) studies have confirmed various pharmacological activities of AP extracts and products. A wide range of pharmacological activities such as antiplatelet aggregation activity [72], immunomodulatory activity [57, 109–113, 139], and other myriad health benefits [10, 75, 132] were observed among these researches. Andrographolide, a major* ent*-labdane diterpenoid of AP, is the largest contributor of many pharmacological activities. Other* ent*-labdane diterpenoids (such as neoandrographolide and 14-deoxyandrographolide), flavonoids, quinic acids, and xanthones are also reported for their significant contributions. A few of the reported works on pharmacology are summarized below.

### 5.1. Effect on Common Cold

*A. paniculata* is commonly used for the prevention and treatment of common cold in several communities. A double-blind, placebo-controlled study of 61 adult patients suffering from common cold used Kan Jang tablets (made from* A. paniculata* dried extract) for 5 days. Within the treatment period, significant clinical improvement was observed on day 4 for 1200 mg extract daily. Both groups showed significant reductions in clinical symptoms like shivering, sore throat, tiredness, muscular ache, rhinitis, sinus pains, and headache [173]. A placebo-controlled study was conducted in 1997 using Kan Jang tablets on 107 healthy students in a rural school as a dose of 2 tablets (200 mg) per day for 3 months to evaluate its efficacy to prevent common cold. The common cold was successfully prevented by Kan Jang tablets with 2.1-fold higher prevention rate compared to the placebo group [174]. Similar successful result was also demonstrated in another study including common cold with sinusitis in another place [175].

### 5.2. Anti-Inflammatory Effect

The anti-inflammatory activity of AP and its bioactive compounds (such as andrographolide and neoandrographolide) has been reported individually by many investigators [28–32, 95–100, 118, 119, 176]. AP extracts and andrographolide showed several anti-inflammatory activities, such as inhibition of intercellular adhesion molecule-1 expression in monocytes activated by tumor necrosis factor-*α* [177], suppression of inducible nitric oxide synthetase (iNOS) in RAW264.7 [29], cyclooxygenase-2 (COX-2) expression in neutrophils and microglial cells [95, 96], reduction of ERK1/2 phosphorylation in murine T cells [90] and IFN-*γ*, and IL-2 production [57, 90], and inhibition of TNF-*α* and GM-CSF induced by LPS [176]. In a study conducted by Hidalgo et al. [96], andrographolide was analyzed for the activation of NF-*κ*B induced by platelet-activating factor (PAF) and N-formylmethionyl-leucyl-phenylalanine (fMLP) in HL-60 cells differentiated into neutrophils. NF-*κ*B controls or contributes to the transcription of more than 200 genes that are involved in a variety of physiological and pathophysiological processes, particularly in immunity and inflammation. It is a critical regulator of cell differentiation, proliferation, and apoptosis and plays pivotal roles in the normal organ development and tumorigenesis [178]. However, the NF-*κ*B can be activated via the signal transduction pathways which are multiple and complex. It was a surprising achievement that andrographolide significantly inhibited the binding of NF-*κ*B to DNA, which is the final step of NF-*κ*B activation and it also decreased the expression of cyclooxygenase-2 (COX-2) by inhibiting the activation of NF-*κ*B in endothelial cells stimulated by PAF [179] and in microglial cells induced by lipopolysaccharide (LPS) [95]. In addition, the kidney inflammation can also be recovered using andrographolide due to its ability to inhibit the NF-*κ*B activated by LPS in kidney [180].

Oral administration of andrographolide at a dose of 300 mg/kg daily had shown significant analgesic activity on acetic-induced writhing in mice and on the Randall-Selitto test in rats, but there was no effect on the hot plate test in mice [78]. Oral administration of andrographolide at 30, 100, and 300 mg/kg also showed anti-inflammatory activity in different models in rats [181]. Iruretagoyena et al. [57] suggested that treatment with andrographolide at a daily dose equal to 4 mg/kg significantly reduced an inflammatory demyelinating disease of the central nervous system, autoimmune encephalitis by inhibiting T cells in mice. The balancing of proinflammatory and anti-inflammatory cytokines is the result of anti-inflammatory performance of andrographolide and regulation of Th1, Th2, and Th17 transcription factors. Andrographolide also increased GATA3 mRNA expression but decreased T-bet and ROR*γ*t mRNA expressions [182].

Besides the AP and andrographolide, andrographolide derivatives (e.g., CHP1002 and andrographolide* sulfonate*) also exhibited anti-inflammatory properties [182, 183]. CHP1002, a novel synthetic derivative of andrographolide, significantly inhibited LPS-induced iNOS, COX-2 expressions, iNOS derived nitric oxide (NO), and COX-2 derived prostaglandin E_2_ (PGE_2_) production through the stimulation of heme oxygenase-1 (HO-1) expression in RAW264.7 macrophages. CHP1002 also significantly faded LPS-stimulated TNF-*α*, IL-1*β*, and IL-6 production [182]. Liu et al. [183] demonstrated that intraperitoneal administration of andrographolide* sulfonate*, a water soluble andrographolide (trade name: Xi-Yan-Ping injection), attenuated the severity of 2, 4, 6-trinitrobenzene sulfonic acid (TNBS) induced colitis in mice. A dose of 1.25–5 mg/kg of andrographolide* sulfonate* significantly recovered the loss of body weights and diarrhea in colitis mice, prevented inflammatory damages of colons and proinflammatory cytokines such as IFN-*γ*, IL-17A, and TNF-*α*, and suppressed the functions of Th1 and Th17 which is a feasible strategy to control inflammatory bowel disease. The demonstrated results suggested that andrographolide* sulfonate* could be a strong therapeutic compound for the treatment of gastrointestinal inflammatory disorders.

### 5.3. Antihyperglycemic Effect

Inhibitions of *α*-glycosidase and *α*-amylase activity and stimulation of insulin sensitivity are considered as effective strategies to lower the level of postprandial blood glucose. These enzymes involved in digestion and absorption of carbohydrates resulting in postprandial increase of blood glucose [184]. Insulin resistance is mainly expressed by hyperinsulinemia and high blood glucose level and is associated with some metabolic hormonal abnormalities, such as dyslipidemia, abnormal uric acid metabolism, increased ovarian testosterone secretion, endothelial dysfunction, elevated procoagulant factors, and elevated inflammatory markers [185]. AP extracts and andrographolide effectively showed antihyperglycemic effect by (a) lowering blood glucose level through inhibition of *α*-glycosidase and *α*-amylase [26, 27, 111, 186]; (b) increasing insulin sensitivity and thus stimulating glucose uptake and oxidation by peripheral tissues [94]; (c) controlling abnormal lipid metabolism; (d) scavenging free radicals from circulation which disrupt the plasma membrane integrity resulting in decreased number of efficient plasma membrane receptors or transporter proteins necessary to uptake glucose from the blood stream [187]. Blood glucose lowering effect of AP was observed in both insulin-lacking diabetic rats and normal rats in several studies [25, 186, 188, 189].

Andrographolide at a dose of 50 mg/kg effectively decreased blood glucose level, stimulated GLUT4 translocation [189], and improved diabetic rat's islet and beta cell functions [190]. Glucose induced hyperglycemia (orally administered) has been prevented by water extracts of AP in nondiabetic rats without affecting epinephrine-induced hyperglycemia [77]. Oral administration of ethanol extracts of AP significantly lowered the fasting blood glucose of human [26, 27, 93, 94]. Another bioactive compound, namely 14-deoxy-11,12-didehydroandrographolide, also showed the antihyperglycemic activity [128].

Besides controlling blood glucose level, andrographolide also effectively prevented the onset of insulitis in a dose dependent manner and thus delayed the onset and suppressed the development of diabetes in 30-week-old NOD mice. Andrographolide also regulates the Th1/Th2/Th17 homeostasis through which it may prevent *β*-cell death and inhibit T-cell infiltration into pancreatic islets and thereby prevent development of type 1 diabetes [191]. Recently, Augustine et al. [187] reported that AP decreases the blood glucose by increasing glucose utilization and oxidation, restoration of insulin signaling molecules in liver, and decreasing the serum lipid levels in high fat and sucrose induced type 2 diabetic rats without showing hypoglycemic effect. A combination of n-hexane insoluble fraction of AP (HIFA) with curcuminoids fraction of* Curcuma xanthorrhiza rhizome* (CFC) also significantly showed the antihyperglycemic effect on high-fructose-fat-fed rats [192]. The combination of HIFA-CFC could be a potential source to develop an antidiabetic agent. Therefore, the identification of more antihyperglycemic compounds of AP and combination of AP with other medicinal plants would be a focusing point of researchers for the better treatment option of the diabetic patients.

### 5.4. Hepatoprotective Effect

*A. paniculata* is widely used traditionally as a hepatoprotective agent and a stimulating agent for multiple enzymes of the liver. It is also used as an ingredient in the polyherbal preparations for the treatment of hepatic disorders in Ayurvedic and Unani medicine [8]. Along with different extracts of AP, andrographolide, neoandrographolide, 14-dexoyandrographolide, and 14-deoxy-11,12-didehydroandrographolide compounds are also reported to have hepatoprotective effect [40–42, 124, 129]. In a comparative study, the leaf extract and andrographolide was tested against the carbon tetrachloride- (CCl_4_-) induced hepatic microsomal lipid peroxidation. Only the leaf extract completely protected the high concentration CCl_4_-induced microsomal lipid peroxidation* in vitro* but not the andrographolide, which indicated that the hepatoprotective role is not solely due to the presence of andrographolide [48]. Similar effect of crude alcohol extracts of the AP leaves against CCl_4_-induced liver damage was also reported by Rana and Avadhoot [50]. Handa and Sharma [40] reported that andrographolide, methanol extract of whole plant, and andrographolide-free methanol extract improved liver histology in rats by 48.6%, 32%, and 15%, respectively, after CCl_4_-induced liver injury. Verma et al. [193] reported the effect of ethanol extract of AP on restoration of different enzyme after CCl_4_-induced liver injury. Further research using specific bioactive compounds is demanding for the better understanding of the hepatoprotective role played by the AP.

### 5.5. Antimicrobial and Antiparasitic Effect

#### 5.5.1. Antibacterial Effect

Modern research has investigated the causes of extensive uses of AP in traditional healing systems as an antimicrobial agent to treat a variety of health morbidities of infectious origin. Leelarasamee et al. [194] reported that crude powder suspended in water to be devoid of* in vitro* antibacterial activity against* Salmonella*,* Shigella*,* Escherichia coli*, gram A* Streptococci*, and* Staphylococcus aureus*, even at a concentration of 25 mg/mL crude powder. However, over the last 3 decades, researchers reported that different types of extracts of* A. paniculata *possess potent antibacterial activity against various pathogenic and nonpathogenic bacteria. Nakanishi et al. [195] reported antibacterial activity of aqueous methanol (50% v/v) crude extracts of whole plant against* Bacillus subtilis* and* Proteus vulgaris*. Although Nakanishi et al. [195] reported the negative result against* E. coli*, ethanol extracts of aerial parts of AP were found to be effective in inhibiting* E. coli* growth along with other ten gram positive and gram negative bacteria species in an investigation conducted by Mishra et al. [196]. The aqueous extract showed significant antibacterial activity due to the combined effect of the isolated andrographolides and arabinogalactan proteins [197]. Similar effect was also mentioned by Fbricant and Farnsworth [198].

The antibacterial activity of three different extracts (dichloromethane, methanol, and aqueous) of AP whole plant was evaluated by Sule et al. [199] against 12 skin infection causing pathogenic bacterial strains. The extracts showed significant effects against all the tested bacterial strains in different concentrations likely 1000, 500, and 250 *μ*g/disc. However, methanol extract showed the highest antibacterial activity against* Enterococcus faecalis* at 1000 *μ*g/disc with an inhibition zone of 24 mm, and dichloromethane extract showed the least activity against* Neisseria meningitis* at 250 *μ*g/disc with an inhibition zone of 6 mm. Similar results again were reported in another manuscript by Sule et al. [200] in the following year. In their study, they investigated antibacterial activity of methanol extract of whole plant against five human pathogenic bacteria* S. aureus*,* Streptococcus pyogenes*,* Micrococcus luteus*,* Proteus mirabilis,* and* P. aeruginosa*. Their results revealed that the highest inhibition (19.67 ± 0.76 mm) was exerted against* S. aureus* at 1000 *μ*g/mL and the least (07.00 ± 1.50 mm) activity shown against* P. aeruginosae* at 250 *μ*g/mL. However, it was a noteworthy result that the methanol extract exhibited more potent inhibitory activity against* S. aureus* (19.67 ± 0.76 mm) and* Streptococcus pyogenes* (16.00 ± 0.58 mm) at 1000 *μ*g/mL compared to the antibiotic vancomycin (17.00 ± 1.05 mm and 14.50 ± 1.00 mm, resp.) [121]. Furthermore, they isolated and characterized two important antibacterial bioactive compounds, namely, 14-deoxyandrographolide (Figure 2) and 3-O-*β*-D-glucosyl-14-deoxyandrographolide, from the whole plant of AP and concluded that the mentioned efficacy of methanol extract had been shown due to the presence of these compounds (Table 4).

#### 5.5.2. Antiviral Effect

Researchers investigated significant antiviral activity of AP besides other pharmacological activities in last two decades. Although they reported antiviral activity against limited viruses, such as dengue virus serotype 1 (DENV-1) [201], human papilloma virus type 16 (HPV16) [202], herpes simplex virus type 1 (HSV-1) [203], influenza A virus [102], and HIV [53, 55, 80, 204], their findings were very encouraging and noteworthy considering the life threatening role of these viruses in human community. The hot aqueous aerial parts extract of AP was reported for its significant antiviral activity to reduce the percentage of HIV antigen-positive H9 cells [55]. Recently, Tang et al. [201] reported that the methanol extract of AP possesses significant inhibition activity against DENV-1* in vitro* assay. Another study has revealed that andrographolide suppressed HPV16 transcription activity, leading to the reduction of E6 oncoprotein and restored p53 [202].

Several bioactive compounds such as andrographolide, neoandrographolide, dehydroandrographolide, natural derivatives of andrographolide, namely, 14-deoxy-11,12-didehydroandrographolide and 14-deoxyandrographolide, and synthetic derivatives, namely, dehydroandrographolide succinic acid monoester (DAMS), 14-*ά*-lipoyl andrographolide (AL-1), 14-acetyl-3,9-isopropyl-ideneandrographolide, 14-acetylandrographolide, 3,14,19-triacetylandrographolide, and 3,9-isopropyl-ideneandrographolide have been shown to have significant antiviral activity against HIV, influenza A, and HSV-1 without any significant cytotoxic effect at virucidal concentrations. Andrographolide, isolated from ethanol extracts of whole plant of AP, showed a great promise in the treatment of HIV infections. It might be able to inhibit viral replication by interfering CDK (cyclin dependent kinase) activity, resulting in deregulation of HIV induced cell cycle [53, 80, 205]. The overall findings of andrographolide effects against different viruses indicate that andrographolide would be an effective agent for prevention and treatment of viral diseases.

#### 5.5.3. Antiparasitic Effect

Antiparasitic activity of the AP extract is reported in certain articles. Dua et al. [143] investigated both* in vitro* and* in vivo* antimalarial activity of four xanthones (Table 4) isolated from roots against* Plasmodium falciparum* and* Plasmodium berghei*. One of the xanthones, 1,2-dihydroxy-6,8-dimethoxy-xanthone, showed substantial antiplasmodial activity during* in vitro* (4 *μ*g/mL at IC_50_ value) and* in vivo* (62% parasitaemia reduction at 30 mg/Kg dose) study.

The water extract of dried leaves was found to be active against adult worms of* Brugia malayi in vitro* [206]. Recently, Padma et al. [207] evaluated the aqueous and methanol extracts for* in vitro* anthelmintic activity against adult earth worms* Pheretima posthuma*. The extracts showed significant results at the concentrations of 25 mg/mL, 50 mg/mL, and 75 mg/mL. However, the clinical relevancies of the antiparasitic studies are inconclusive due to obtaining the results at high concentration that may not be feasible clinically.

### 5.6. Anticancer Effect

Andrographolide exhibited both direct and indirect effects on cancer cells by inhibiting proliferation of cancer cells, cell-cycle arrests, or cell differentiation, enhancing body's own immune system against cancer cells; and inducting apoptosis and necrosis of cancer cells [208]. Dichloromethane fraction of methanol extract significantly inhibited the proliferation of HT-29 colon cancer cells. The major bioactive compound of AP, andrographolide, isolated from dichloromethane inhibited the growth of a diverse cancer cell representing different types of human cancers [11]. In contrast, recently Aditya et al. [209] reported that methanol extract of AP was found to be very less effective against both MCF-7 breast and HT-29 colon cancer cell lines. This low activity exhibited might be due to the low penetration power of the active principles.

Antiproliferative activities of andrographolide and isoandrographolide along with other 16* ent*-labdane diterpenoids isolated from 85% ethanol extract of AP against human leukaemia HL-60 cells have also been investigated by Chen et al. [138]. These results showed that andrographolide and isoandrographolide were more effective than others. In a recent study, Chen et al. [142] identified a new flavonoid, 7, 8-dimethoxy-2′-hydroxy-5-O-*β*-d-glucopyranosyloxyflavone, isolated from the aerial parts of AP. This flavonoid exhibited potent antiproliferative activity against human leukaemia HL-60 cells with IC_50_ of 3.50 *μ*M. Ethanol (70%) extracts and andrographolide were also found to be effective to increase the life spans of thymoma injected mice cells in an* in vivo* study [83]. In the following year, Geethangili et al. [210] showed the effective cytotoxic activity of ethanol extracts against human cancer cells including Jurkat (lymphocytic), PC-3 (prostate), HepG2 (hepatoma), and colon 205 (colonic) cancer cells. In another study, use of andrographolide at a dose 12 *μ*g/mL for 36 h against HL-60 cells improved 27% in G0/G1 phase cells and significantly decreased cells number at S and G2/M phase [12]. Shi et al. [82] reported that andrographolide can inhibit human colorectal carcinoma (CRC) Lovo cell growth by G1–S phase arrest and induce the expression of cell-cycle inhibitory proteins p53, p21, and p16. These proteins repressed the activity of cyclin D1/Cdk4 and/or cyclin A/Cdk2, required for G1 to S phase transition. In a recent* in vitro* study, andrographolide has been shown to suppress the growth and invasion of CRC Lovo cells and trigger apoptosis. Besides the effect of andrographolide alone, andrographolide in combination with chemotherapeutics, cisplatin, is likely to represent a potential therapeutic strategy for CRC [211].

Andrographolide and its analogues exert direct inhibitory effect on cancer cells by inducing expression of cell cycle inhibitory proteins and depressing cyclin-dependent kinase (Cdk) resulting in blocking the cell cycle progression at G0/G1 [12, 13, 82, 212, 213]. Some other compounds also block the cell cycle progression at G2/M phase [210]. A novel semisynthetic analogue of andrographolide, DRF3188, exhibited anticancer activities against MCF 7 breast cancer cells at a lower dosage than andrographolide through similar mechanism [212]. Both the compounds block cell cycle at the G0-G1 phase through induction of the cell cycle inhibitor (p27) and concomitant decrease in the levels of Cdk4. Therefore, attention has been focused on the anticancer properties of pure components of AP and the molecular target of andrographolide that blocks G1 stage still needs to be determined.

### 5.7. Immunomodulatory Effect

Control of immune response by regulating nuclear factor of activated T cells (NFAT), a transcription factor essential for cytokine production during T-cell activation, is a widely known strategy. Preventing translocation of NFAT to nucleus is the target of several immunosuppressive agents (e.g., cyclosporin A, FK506) [112]. AP is known to exert several immunomodulatory properties. More than two decades ago, a laboratory test demonstrated that AP inhibited growth of human breast cancer cells similar to the drug tamoxifen [17]. Amroyan et al. [214] reported that andrographolide was effective to stop the clumping of blood platelets that lead to heart attacks and they also suggested that andrographolide has a major effect on activating the general defense functions of immune system by stimulating the production of antibodies as well as nonspecific immune responses such as increased macrophage phagocytosis. An* in vitro* study with the increased proliferation of lymphocytes and production of interleukin-2 (IL-2) confirmed the immunostimulatory activity of AP [12]. Three diterpene compounds of AP isolated from dichloromethane fraction of methanol extract showed augmented proliferation and IL-2 induction in human peripheral blood lymphocytes (HPBLs) at a low concentration [11]. In addition, the chronic consumption of the aqueous extract of AP also promoted the immune functions at 250 mg/kg and 500 mg/kg; however, 1000 mg/kg dose leading to development of autoimmune reactions, anaemia and multiple myeloma [215]. AP extract and andrographolide significantly promoted the lyses of natural killer (NK) cell-mediated target cells on day 5 after tumor induction. Antibody dependent cell mediated cytotoxicity (ADCC) and antibody dependent complement-mediated cytotoxicity (ACC) in metastatic tumor bearing animals were also enhanced significantly compared to the control by the treatment of AP extract and andrographolide. In addition, the levels of proinflammatory cytokines such as IL-1*β*, IL-6, GM-CSF, and TNF-*α* were also effectively reduced [216]. Carretta et al. [112] demonstrated that andrographolide reduces IL-2 production, extracellular signal regulated kinase- (ERK-) 1 and ERK-5 phosphorylation induced by anti-CD3 or phorbol myristate acetate and ionomycin (PMA/Ionomycin), and NF-*κ*B activity in Jurkat cells and this effect can be related to a reduction in NFAT activity and an increase in c-jun-N-terminal kinase (JNK) phosphorylation. Moreover, andrographolide inhibited tumor growth in animals by stimulating the production of cytotoxic T lymphocytes [83]. Hence, the compounds modulate the host immune systems against these cells to confer the direct cytotoxicity to cancer cells. Based on the reported immunomodulatory properties, AP and andrographolide might be effective clinically to treat the autoimmune diseases.

### 5.8. Cardiovascular Effect

Cardiovascular diseases (CVDs) are the leading cause of death throughout the world. AP is used widely for improving the cardiac health in traditional medicinal systems. Several studies have investigated its activities in cardiovascular diseases [38, 81, 107, 131, 217–225]. Wang et al. [222] reported that AP is potential to increase the nitric oxide, cyclic guanosine monophosphate, and superoxide dismutase activity with declines of lipid peroxide and endothelin in an atherosclerotic rabbit model. In another study, Wang and Zhao [223] also investigated that the AP extracts can be able to prevent constriction of blood vessels and increase blood clotting time significantly in the pre- and postangioplasty procedures. The extracts inhibited the cell growth and DNA synthesis in a dose dependent manner, which is a similar mechanism like stents that prevent cell division.

Aqueous extracts and active constituents of AP showed significant antihypertensive activity in both spontaneously hypertensive rats and normotensive Wister-Kyoto rats [226], improved the blood pressure status in both pre- and post experimental myocardial infarction in animals [217, 218], and exhibited platelet antiaggregation in* in vitro* [81, 214] and* ex vivo* [227] assays. The existing reports suggested that AP can be used as alternative source of the treatment of CVDs. Further studies are necessary to know the insight of the mechanism of actions of specific constituents of AP and in clinical perspectives.

### 5.9. Antihyperlipidemic Effect

Hyperlipidemia is an important factor for atherosclerosis that leads to heart attack (obstruction occurs in the coronary arteries) and stroke (obstruction occurs in the arteries of the brain) [228–230]. Chen et al. [231] suggested the andrographolide as candidate therapeutic agent for atherosclerosis based on their research result. Recently, another study revealed antihyperlipidemic effects of andrographolide and neoandrographolide [225]. Yang et al. [225] reported the effects of andrographolide and neoandrographolide on hyperlipidemic mice induced by 75% yolk emulsion and hyperlipidemic rats induced by high fat emulsion. Andrographolide and neoandrographolide reduced triglyceride, total cholesterol, and low-density lipoprotein cholesterol in a dose dependent manner. Plasma aspartate transaminase and alanine transaminase levels were also significantly (*P* < 0.01) reduced compared to the positive control (Simvastatin). These compounds exhibited their lipid and lipoprotein reducing effects through downregulation of iNOS expression and upregulation of eNOS expression in aorta of hyperlipidemic rats [225]. The results of hypolipidemic study require further exploration of the molecular mechanism and the related signaling pathway.

### 5.10. Sexual Functions and Contraceptive Effect

AP and andrographolide showed earlier either contraceptive [58, 232–236], fertility [237–239], or no effects [240] in a variety of studies. AP showed contraceptive effects by terminating spermatogenesis in male albino rats [233, 236]. Zoha et al. [232] reported that there were no pregnant female mice that consumed AP mixed food daily after mating with the untreated male of potential fertility, which means that the AP has contraceptive effect on female mice. AP and andrographolide were effective to prevent cytokinesis of dividing spermatogenic cell lines resulting in stopped spermatogenesis [235, 236]. The antifertility effects of andrographolide are characterized by marked decreases of protein content along with significant increases of cholesterol, acid phosphatase, and alkaline phosphatase levels with appearance of fructose in the reproductive systems of rats [234]. Panossian et al. [241] reported negative results of AP's powdered extract on blood progesterone content in rats. However, the testosterone level with mounting frequency was significantly increased in mice at 4 weeks of andrographolide treatment. There was no toxicity of andrographolide (50 mg/kg) treatment for up to 8 weeks on number and motility of sperm. Further studies are needed to confirm the double stand activities of AP and andrographolide on female and male sexual behaviors. The investigation of specific mechanisms in regulation of fertility and contraceptive effects of AP and its active constituents would be valued to identify target point of controlling sexual behaviors.

### 5.11. Safety and Toxicity Effects

Generally, uses of AP as a medicine have been proved to be safe in various studies on mice, rats, and rabbits, as well as in* in vitro* assays and some clinical trials. Some conflicting results are also available. Few studies showed the toxic effect of AP on reproductive system by damaging the Sertoli cell in male gonads in albino rats. A dose of 25 and 50 mg/kg body weight for a period of 48 days demonstrated that antispermatogenic effect [58, 235, 236]. However, contradictory result was also demonstrated by numerous studies [237, 239, 240, 242–245]. The safety of AP extracts regarding the oral acute toxicity (>17 g/Kg, LD_50_) [237], testicular toxicity (>1 g/Kg, LD_50_) [240], and genotoxicity (5 g/Kg, LD_50_) [244] has been reported.

Due to AP's extreme bitterness, it may cause emesis. Some adverse effects including allergic reaction, gastric instability, fatigue, headache, loss of appetite, lymphadenopathy, diarrhea, metallic taste, and nausea are also observed in overdosing of AP extracts [63, 246]. It is suggested to avoid this plant during pregnancy due to ovulation preventive effects of the plant [232]. To date, all trials with few exceptions were for short duration; thus the prediction of safety for long term use would be farfetched.

## 6. Future Direction

Pharmacological activity of AP was investigated either using crude extracts or isolated bioactive compounds. Though the crude extract showed significant effects, isolation of bioactive compounds and investigation of pharmacology provide more specific knowledge especially about mechanism of actions of compounds. Conventional extraction processes (such as soxhlet extraction, maceration, and hydrodistillation) have been using by most of the researchers all over the world. However, selection of proper extraction methods is crucial for qualitative and quantitative studies of bioactive compounds derived from medicinal plant [247].

The reported pharmacology of bioactive compounds of AP is based on conventional extraction methods with few exceptions using different solvents, for example, methanol, ethanol, water, acetone, acetone-water, chloroform, and dichloromethane (Table 4). The conventional extraction techniques have some limitations including longer extraction time, costly and high purity solvent required, huge amount of solvent evaporation, low extraction selectivity, and thermal decomposition of thermolabile compounds. To overcome these problems, some new and promising extraction methods like ultrasound assisted extraction, enzyme-assisted extraction, microwave-assisted extraction, pulsed electric field assisted extraction, supercritical fluid extraction, and pressurized liquid extraction have been introduced which are termed as nonconventional extraction methods [248]. Some of these techniques are known as “green techniques” and are applicable for high yield and more purified compounds within a short time compared to classical methods. For example, extraction of phenolic antioxidants from five citrus peels (Yen Ben lemon, Meyer lemon, grapefruit, mandarin, and orange) by enzyme-assisted extraction was improved significantly with higher enzyme concentration [249]. Howard and Pandjaitan [250] extracted flavonoids from spinach by pressurized liquid extraction effectively. Therefore, it can be suggested that extraction of bioactive compounds from AP using nonconventional extraction methods of bioactive compounds would be valued to get high yield and purified compounds.

We mentioned earlier that tissue culture techniques have been applied successfully to form new flavones by differentiating callus culture. Jalal et al. [168] reported that differentiating tissue cultures of AP produce three new flavones, 5-hydroxy-7,8,2′-trimethoxy-, 5,2′-dihydroxy-7,8-dimethoxy-, and 5-hydroxy-7,8-dimethoxy-flavones. Arifullah et al. [141] isolated andrographolide and echiodinin from* in vitro* leaf callus of AP.* In vitro* root culture system can be exploited as a renewable source of minerals essential for designing effective drugs [167]. Other bioactive compounds can also be extracted via tissue culture. Adventitious roots from vegetative propagation of AP can also be an alternative source of plant materials for extraction of bioactive compounds from roots. We investigated a rapid and maximum adventitious roots in AP's shoot microcuttings applying soaking method at 3 mM IBA (Indole-3-butyric acid) (Figure 1(f)) (unpublished data). These adventitious roots can be further investigated to see the quality and content of bioactive compounds and pharmacological effects. Hence, adventitious rooting and plant tissue culture techniques can be alternative ways to meet the commercial demand of AP.

Stress alters growth and development of plants. In our investigation, we found that salinity stress showed deleterious effects on morphophysiological parameters including colour, plant height, leaf area, and root length of AP. It also causes less production of AP [251]. The stress conditions have a strong impact on the responsible metabolic pathways for the accumulation of the related natural products [252]. However, abiotic stress condition (as an agronomical approach) [253, 254] and biotechnological approaches (genetic transformation) [255] can be used to improve the content of some active compounds like flavonoids on a large scale in plants. Talei et al. [256] reported that high salinity level (16 dS/m) reached relatively higher andrographolide and neoandrographolide content compared to control. The increase in andrographolide and neoandrographolide contents at high salinity level (16 dS/m) was 177.57% and 131.18% compared to the control, respectively. Optimization of the yield of andrographolide and neoandrographolide content and investigation of other important bioactives of AP under abiotic stresses would be valued to meet the commercial demand of bioactive compounds of AP. To date, to our knowledge, any investigation of pharmacological activities of bioactive compounds obtained from salinity treated AP is not conducted yet. Therefore, it is very crucial to know the comparative efficacy of the compounds obtained from salinity treated and control AP. The obtained knowledge from the suggested study could also be used for further research design on AP for the betterment of human health.

## 7. Conclusion

The demand of AP is greatly increased in the past few years for its overwhelming therapeutic potentials. Available data on AP also clearly expresses a broad spectrum of pharmacological properties of this plant. Due to possessing extensive pharmacological activities, the AP can be safely regarded as one of the modern catholicons. However, the investigated pharmacological activities of AP need validation through the clinical study. Though several clinical studies were successfully completed without adverse effects or fatalities, most of them only investigated upper respiratory tract infections for a variety of conditions. Verification of the efficacy of other biological activities of AP including antidiabetic, anticancer, anti-inflammatory, and hepatoprotective activities, on human study subjects would bring a lot of benefits for the largest population of the globe. We assume that the AP could be useful as highly applied therapeutic agents for a variety of disorders in the near future to cure human diseases as well as some animal diseases. To fulfill this dream, the researchers might focus on multiplication of this plant to meet commercial demand besides the pharmacology study. Tissue culture techniques might be a good alternative to make AP available for researches (i.e., pharmacological study and phytochemical study to find new bioactive compounds) as well as conservation of this plant.

## Figures and Tables

**Table 1:** The morphology and physiology of *A. paniculata* [10, 62, 63].

Traits	Values/characteristics
Plant height	30–110 cm
Stem	Dark green
Length	30–100 cm
Diameter	2–6 mm
Shape	Quadrangular with longitudinal furrows and wings on the angles of the young parts, slightly enlarged at the nodes
Leaves	Glabrous
Length	2–12 cm
Width	1–3 cm
Arrangement	Lanceolate
Shape	Pinnate, acute apex, entire margin
Flowers	White with rose-purple spots on the petals
Size	Small, in lax spreading axillary and terminal racemes or panicles
Seed	Capsules linear-oblong, acute at both ends
Size	1.9 cm × 0.3 cm
Color	Yellowish brown
Shape	Subquadrate, numerous
Flowering and fruiting	December to April

**Table 2:** The vernacular names of *A. paniculata* [10, 71, 75, 76].

Language	Name
Arabic	Quasabhuva
Assamese	Chiorta, Kalmegh
Azerbaijani	Acılar Şahı, Acılar Xanı (khanı)
Bengali	Kalmegh
Burmese	Se-ga-gyi
Chinese	Chuan Xin Lian
English	The Creat, King of Bitters
French	Chirette verte, Roi des amers
Gujarati	Kariyatu
Hindi	Kirayat, Kalpanath,
Indonesian	Sambiroto, Sambiloto
Japanese	Senshinren
Kannada	Nelaberu
Konkani	Vhadlem Kiratyem
Lao	La-Sa-Bee
Malay	Hempedu Bumi, Sambiloto
Malayalam	Nelavepu, Kiriyattu
Manipuri	Vubati
Marathi	Oli-kiryata, Kalpa
Mizo	Hnakhapui
Oriya	Bhuinimba
Panjabi	Chooraita
Persian	Nain-e Havandi
Philippines	Aluy, Lekha and Sinta
Russian	Andrografis
Sanskrit	Kalmegha, Bhunimba and Yavatikta
Scandinavian	Green Chiratta
Sinhalese	Hīn Kohomba or Heen Kohomba
Spanish	Andrografis
Tamil	Nilavembu
Telugu	Nilavembu
Thai	Fa-Talai-Jorn, Fah-talai-jon (jone)
Turkish	Acılar Kralı, Acı Paşa, Acı Bey
Urdu	Kalmegh, Kariyat, Mahatita
Vietnamese	Xuyên Tâm Liên

**Table 3:** The ethnobotanical uses of *A. paniculata*.

Country/TMS^*^	Traditional uses	Reference
Ayurvedic	Fever, liver diseases, torpid liver, vitiligo	[62]

Japan	Fever, common cold	[10]

Malaysia	Diabetes, hypertension	[10, 77]

Scandinavian	Fever, common cold	[10]

Traditional Bangladeshi medicine	Acute diarrhea, anorexia, bloating with burning sensations in the chest, blood purifier, common cold, constipation, cough, debility, diabetes, dysentery, edema, emesis, fever, headache, helminthiasis, indigestion, intestinal worms, leucorrhea, liver disorders, loss of appetite, low sperm count, lower urinary tract infections, lung infections, malaria, mucus, pharyngotonsillitis, sexual and skin disorders, splenomegaly, uncomplicated sinusitis, vertigo	[9]

Traditional Chinese medicine	Inflammation, fever, burn, carbuncle, cervical erosion, chicken pox, common cold, cough with thick sputum, detoxicant, detumescent, diarrhea dispel toxins of the body, dysentery, eczema, epidemic encephalitis B, fever, hepatitis, herpes zoster, laryngitis, mumps, neonatal subcutaneous annular ulcer, neurodermatitis, pelvic inflammation, pharyngitis, pharyngolaryngitis, pneumonia, respiratory infections, snake bites, sores, suppurative otitis media, tonsillitis, vaginitis	[8, 10]

Traditional Indian medicine	Diabetes, dysentery, enteritis, helminthiasis, herpes, peptic ulcer, skin infections (topical use), snake-bites (topical use)	[10]

Traditional Thai medicine	Fever, common cold, noninfectious diarrhea	[10]

Unani system of medicine	Anthelmintic, anti-inflammatory, antipyretic, aperient, astringent, boils, carminative, chronic and seasonal fevers, convalescence after fevers, diuretic, dysentery, dyspepsia associated with gaseous distension, emmenagogue, emollient, gastric and liver tonic, general debility, gonorrhea, irregular bowel habits, leprosy, loss of appetite, relieve griping, scabies, skin eruptions	[8]

^*^Traditional Medicinal Systems.

**Table 4:** Preclinical pharmacology of isolated phytoconstituents of *A. paniculata*.

">		
Compound 3D/2D, chemical properties/IUPAC name/part used/extracts/pharmacology		

*ent*-labdane diterpenoids		

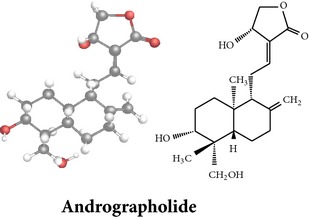	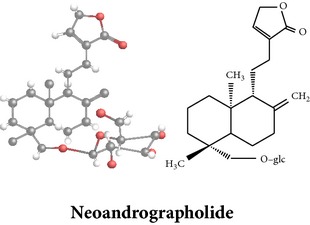	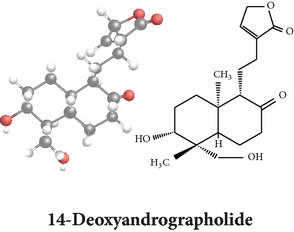
Chemical properties: C_20_H_30_O_5_ Exact mass: 350.21; Mol. Wt.: 350.45; C, 68.54; H, 8.63; O, 22.83 IUPAC: (3E,4S)-3-[2-[(1R,4aS,5R,6R,8aS)-6-hydroxy-5-(hydroxymethyl)-5,8a-dimethyl-2-methylidene-3,4,4a,6,7,8-hexahydro-1H-naphthalen-1-yl]ethylidene]-4-hydroxyoxolan-2-one Part used: L^$,§,¢,^, AeP^∗,#,¤,œ,†,*£*,*ð*^, WP^£,ø^, R^*¥*^ Extracts: M^§,¤,¢,*£*,*¥*,ø,$,*ð*^, E^∗,#,$^, H^œ^, AW^†^ Pharmacology: analgesic, antipyretic, antiulcerogenic [78], antiangiogenic [79], antiretroviral [33, 80], antithrombotic [81], anticancer [13, 16, 82–91], antidiabetic [25–27, 92–94], anti-inflammatory [95–100], anti-influenza [101, 102], antileishmaniasis [103], antiproliferative, proapoptotic [104, 105], antiurothelial [106], cardioprotective [107], cholestatic [108], hepatoprotective [40, 41], immunomodulatory [57, 90, 109–114], inhibition of Epstein Barr virus [115], protective activity against alcohol-induced hepatic, radiosensitiser [116], renal toxicity [117]	Chemical properties: C_26_H_40_O_8_ Exact mass: 480.27; Mol. Wt.: 480.59; C, 64.98; H, 8.39; O, 26.63 IUPAC: 4-[2-[(4aS,5R,8aS)-5,8a-dimethyl-2-methylidene-5-[[(2R,3R,4S,5S,6R)-3,4,5-trihydroxy-6-(hydroxymethyl)oxan-2-yl]oxymethyl]-3,4,4a,6,7,8-hexahydro-1H-naphthalen-1-yl]ethyl]-2H-furan-5-one Part used: L^$,§,¢,^, WP^£,ø^, R^*¥*^, AeP^∗,¤,*¥*,*ð*,†^ Exacts: M^§,¤,¢,*£*,*¥*,ø,*ð*^, E^∗,$^, AW^†^ Pharmacology: antiherpes-simplex virus [33], anti-inflammatory [100, 118, 119], antioxidant [35], antiparasitic [34], chemosensitiser [120], hepatoprotective [9, 43]	Chemical properties: C_19_H_28_O_5_ Exact mass: 336.19; Mol. Wt.: 336.42; C, 67.83; H, 8.39; O, 23.78 IUPAC: 3-(2-((1R,2R,4aR,5S,8aS)-decahydro-2-hydroxy-1-(hydroxymethyl)- 1,4a-dimethyl-6-oxonaphthalen-5-yl)ethyl)furan-2(5H)-one Part used: L^§,$,¢,‡^, WP^*£*,©,*®*,ø^, AeP^∗,¤,*¥*,#,*ð*,œ,†^ Extracts: M^§,¤,¢,ø,©,*ð*^, E^∗,#,$^, H^œ^, AW^†^ Pharmacology: antibacterial [121, 122], antifungal [123], hepatoprotective [124], immunomodulator [113], platelet activating factor antagonist [110], uterine smooth muscle relaxant [125], vasorelaxant, antihypertensive [126, 127]

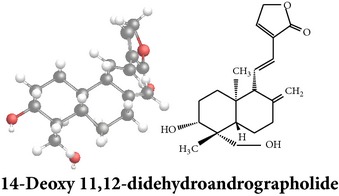	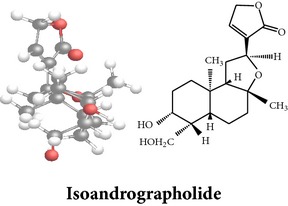	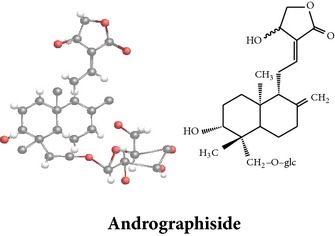
Chemical properties: C_20_H_28_O_4_ Exact mass: 332.2; Mol. Wt.: 332.43; C, 72.26; H, 8.49; O, 19.25 IUPAC: 4-[(E)-2-[6-hydroxy-5-(hydroxymethyl)-5,8a-dimethyl-2-methylidene-3,4,4a,6,7,8-hexahydro-1H-naphthalen-1-yl]ethenyl]-2H-furan-5-one Part used: L^§,$,¢,‡^, WP^*£*,©,*®*,ø^, AeP^∗,¤,*¥*,#,*ð*,œ,†^ Extracts: M^∗,#,§,¤,*£*,*¥*,*®*,ø,*ð*^, E^$^, H^œ^, DCM^®^ Pharmacology: antidiabetic [128], antifungal [123], antioxidant, hepatoprotective [129], antiretroviral [80], antithrombotic [81], cholestatic [108], cytotoxic [130], vasorelaxant, antihypertensive [126, 127, 131], antiherpes [33], vasorelaxant, antihypertensive [126]	Chemical properties: C_19_H_28_O_5_ Exact mass: 336.19; Mol. Wt.: 336.42; C, 67.83; H, 8.39; O, 23.78 IUPAC: 3-((2S,3aR,5aR,6R,7R,9aR,9bS)-dodecahydro-7-hydroxy-6-(hydroxymethyl)-3a,9a-dimethylnaphtho[2,1-b]furan-2-yl)furan-2(5H)-one Part used: L^¢,^, AeP^#,¤,*¥*,†^, WP^ø^, R^*¥*^ Extracts: M^¤,¢,*¥*,ø^, E^#^, AW^†^ Pharmacology: anti-inflammatory and anticancer [132], antiproliferative [133], cytotoxic [14], differentiation inducer [18], inhibits growth of *Bacillus subtilis* [134]	Chemical properties: C_27_H_42_O_10_ Exact mass: 526.28; Mol. Wt.: 526.62; C, 61.58; H, 8.04; O, 30.38 IUPAC: NA Part used: L^§,$,¢,‡^, WP^*£*,©,*®*,ø^, AeP^∗,¤,*¥*,#,*ð*,œ,†^ Extracts: M^¤,*£*,‡,*ð*^, E^∗,#,$^, AW^†^, PtE^‡^, CHL^‡^ Pharmacology: hepatoprotective [42]

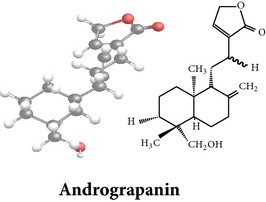	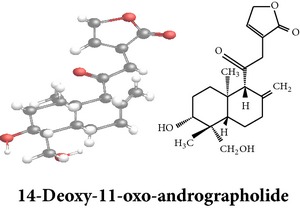	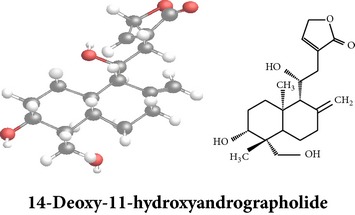
Chemical properties: C_20_H_30_O_3_ Exact mass: 318.22; Mol. Wt.: 318.45; C, 75.43; H, 9.50; O, 15.07 IUPAC: 3-(2-((1R,4aS,5R)-decahydro-1-(hydroxymethyl)-1,4a-dimethyl-6-methylenenaphthalen-5-yl)ethyl)furan-2(5H)-one Part used: L^§,æ^, AeP^*β*,œ,†^ Extracts: E^*β*,æ^, H^œ^ Pharmacology: anti-inflammatory [135, 136]	Chemical properties: C_20_H_28_O_5_ Exact mass: 348.19; Mol. Wt.: 348.43; C, 68.94; H, 8.10; O, 22.96 IUPAC: 3-(2-((1R,2R,4aR,5R,8aS)-decahydro-2-hydroxy-1-(hydroxymethyl)-1,4a-dimethyl-6-methylenenaphthalen-5-yl)-2-oxoethyl)furan-2(5H)-one Part used: L^§,æ^, AeP^*β*,œ,†^ Extracts: M^†^, AW^§^ Pharmacology: antileishmaniasis [137]	Chemical properties: C_20_H_30_O_5_ Exact mass: 350.21; Mol. Wt.: 350.45; C, 68.54; H, 8.63; O, 22.83 IUPAC: NA Part uses: AeP^¤,†^, WP^*£*^ Extracts: M^¤,*£*^, AW^†^ Pharmacology: cell differentiation inducer [18]

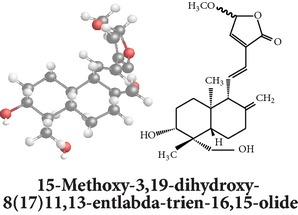	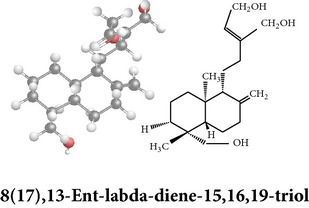	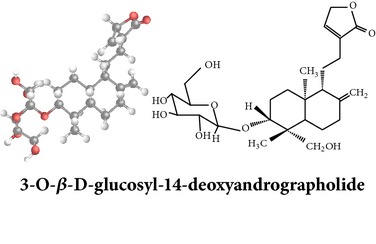
Chemical properties: C_21_H_30_O_5_ Exact mass: 362.21; Mol. Wt.: 362.46; C, 69.59; H, 8.34; O, 22.07 IUPAC: 3-((E)-2-((1R,2R,4aR,5R,8aS)-decahydro- 2-hydroxy-1-(hydroxymethyl)-1,4a- dimethyl-6-methylenenaphthalen-5-yl)vinyl)-5-methoxyfuran-2(5H)-one Part used: AeP Extracts: E^#^ Pharmacology: antiproliferative [138]	Chemical properties: C_20_H_34_O_3_ Exact mass: 322.25; Mol. Wt.: 322.48; C, 74.49; H, 10.63; O, 14.88 IUPAC: (Z)-2-(2-((1R,4aS,5R,8aS)-decahydro-1-(hydroxymethyl)-1,4a-dimethyl-6-methylenenaphthalen-5-yl)ethyl)but-2-ene-1,4-diol Part used: AeP Extracts: E^#^ Pharmacology: antiproliferative [138]	Chemical properties: C_26_H_40_O_9_ Exact mass: 496.27; Mol. Wt.: 496.59; C, 62.88; H, 8.12; O, 29.00 IUPAC: 3-(2-((1R,2R,4aS,5R)-decahydro-1-(hydroxymethyl)-1,4a-dimethyl-6-methylene-2-(tetrahydro-3,4,5-trihydroxy-6-(hydroxymethyl)-2H-pyran-2-yloxy)naphthalene-5-yl)ethyl)furan-2(5H)-one Part used: AeP^þ^, WP^®^ Extracts: M^®^, E^þ^ Pharmacology: antibacterial [121, 122], antifungal [123]

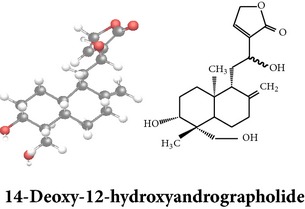	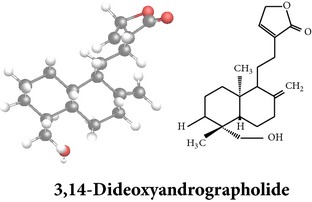	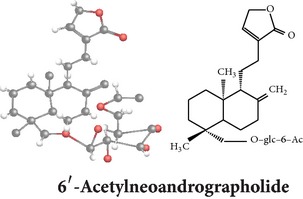
Chemical properties: C_20_H_30_O_5_ Exact mass: 350.21, Mol. Wt.: 350.45, C, 68.54; H, 8.63; O, 22.83 IUPAC: NA Part used: AeP Extracts: M^¤^, AW^†^ Pharmacology: antimicrobial [134]	Chemical properties: C_20_H_30_O_3_ Exact mass: 318.22, Mol. Wt.: 318.45; C, 75.43; H, 9.50; O, 15.07 IUPAC: 3-(2-((1R,4aS,8aS)-decahydro-1-(hydroxymethyl)-1,4a-dimethyl-6-methylenenaphthalen-5-yl)ethyl)furan-2(5H)-one Part used: AeP Extracts: E^#^ Pharmacology: antiproliferative [138]	Chemical properties: C_28_H_44_O_8_ Exact mass: 508.3, Mol. Wt.: 508.64; C, 66.12; H, 8.72; O, 25.16 IUPAC: 3-(2-(1-(((2R)-6-(ethoxymethyl)-tetrahydro-3,4,5-trihydroxy-2H-pyran-2-yloxy)methyl)-decahydro-1,4a-dimethyl- 6-methylenenaphthalen-5-yl)ethyl)furan-2(5H)-one Part used: AeP Extracts: M^¤^ Pharmacology: cell differentiation inducer [18]

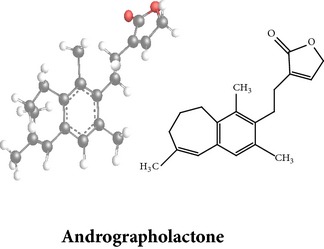	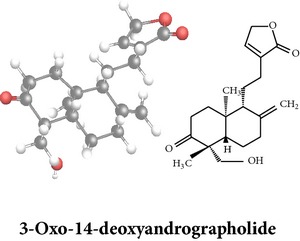	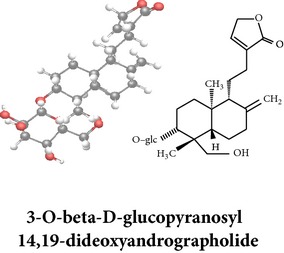
Chemical properties: C_20_H_24_O_2_ Exact mass: 508.3; Mol. Wt.: 508.64; C, 66.12; H, 8.72; O, 25.16 IUPAC: 3-(2-(1-(((2R)-6-(ethoxymethyl)- tetrahydro-3,4,5-trihydroxy-2H-pyran-2-yloxy)methyl)-decahydro-1,4a-dimethyl-6-methylenenaphthalen-5-yl)ethyl)furan-2(5H)-one Part used: AeP Extracts: E^¬^ Pharmacology: cytotoxic [139]	Chemical properties: C_20_H_28_O_4_ Exact mass: 332.2; Mol. Wt.: 332.43; C, 72.26; H, 8.49; O, 19.25 IUPAC: 3-(2-((1R,4aS,5R,8aS)-decahydro-1-(hydroxymethyl)-1,4a-dimethyl-6-methylene-2-oxonaphthalen-5-yl)ethyl)furan-2(5H)-one Part used: AeP Extracts: E^#^ Pharmacology: antiproliferative [138]	Chemical properties: C_26_H_40_O_9_ Exact mass: 496.27; Mol. Wt.: 496.59; C, 62.88; H, 8.12; O, 29.00 IUPAC: 3-(2-((1R,2R,4aS,5R,8aS)-decahydro-1-(hydroxymethyl)-1,4a-dimethyl-6-methylene-2-(tetrahydro-3,4,5-trihydroxy-6-(hydroxymethyl)-2H-pyran-2-yloxy)naphthalen-5-yl)ethyl)furan- 2(5H)-one Part used: AeP Extracts: AW^†^ Pharmacology: antimicrobial [134]

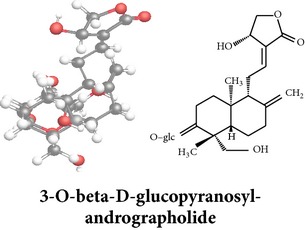	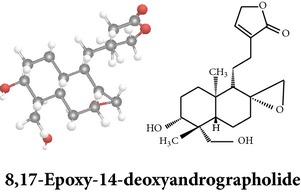	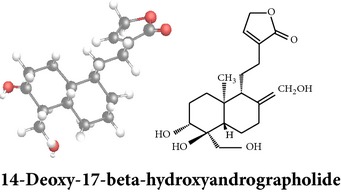
Chemical properties: C_26_H_40_O_10_ Exact mass: 512.26; Mol. Wt.: 512.59; C, 60.92; H, 7.87; O, 31.21 IUPAC: (S,E)-dihydro-3-(2-((1R,2R,4aS,5R,8aS)-decahydro-1-(hydroxymethyl)-1,4a-dimethyl-6-methylene-2-(tetrahydro-3,4,5- trihydroxy-6-(hydroxymethyl)-2H-pyran-2-yloxy)naphthalen-5-yl)ethylidene)-4-hydroxyfuran-2(3H)-one Part used: AeP Extracts: AW^†^ Pharmacology: antimicrobial [134]	Chemical properties: C_20_H_32_O_5_ Exact mass: 352.22; Mol. Wt.: 352.47; C, 68.15; H, 9.15; O, 22.70 IUPAC: NA Part used: AeP Extracts: AW^†^ Pharmacology: antimicrobial [134]	Chemical properties: C_20_H_32_O_5_ Exact mass: 352.22; Mol. Wt.: 352.47; C, 68.15; H, 9.15; O, 22.70 IUPAC: 3-(2-((1R,2R,4aS,5R,8aS)-decahydro-2-hydroxy-1,6-bis(hydroxymethyl)-1,4a-dimethylnaphthalen-5-yl)ethyl)furan-2(5H)-one Part used: AeP Extracts: AW^†^ Pharmacology: antimicrobial [134]

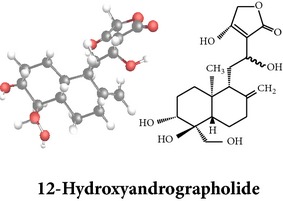	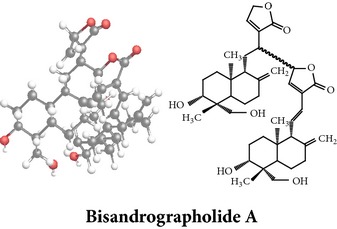	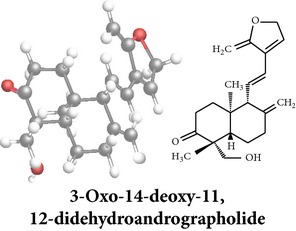
Chemical properties: C_19_H_28_O_7_ Exact mass: 368.18; Mol. Wt.: 368.42; C, 61.94; H, 7.66; O, 30.40 IUPAC: NA Part used: AeP Extracts: E^#^ Pharmacology: antiproliferative [138]	Chemical properties: C_40_H_56_O_8_ Exact mass: 664.4; Mol. Wt.: 664.87; C, 72.26; H, 8.49; O, 19.25 IUPAC: NA Part used: AeP Extracts: M^¤^ Pharmacology: cell differentiation inducer [18], transient receptor potential channel vanilloid-4 (TRPV-4), activator/analgesic, anti-inflammatory [140]	Chemical properties: C_21_H_28_O_3_ Exact mass: 328.2; Mol. Wt.: 328.45; C, 76.79; H, 8.59; O, 14.61 IUPAC: (1R,4aR,5R,8aS)-octahydro-5-((1E)-2-(2,5-dihydro-2-methylenefuran-3-yl)vinyl)-1-(hydroxymethyl)-1,4a-dimethyl-6-methylenenaphthalen-2(1H)-one Part used: AeP Extracts: E^#^ Pharmacology: antiproliferative [138]

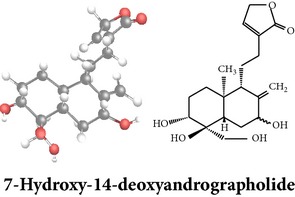	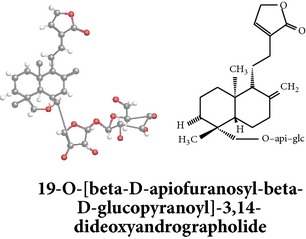	
Chemical properties: C_19_H_28_O_6_ Exact mass: 352.19; Mol. Wt.: 352.42; C, 64.75; H, 8.01; O, 27.24 IUPAC: 3-(2-((1S,2R,4aS,5S,8aS)-decahydro-1,2,7-trihydroxy-1-(hydroxymethyl)-4a-methyl-6-methylenenaphthalen-5-yl)ethyl)furan- 2(5H)-one Part used: AeP Extracts: E^#^ Pharmacology: antiproliferative [138]	Chemical properties: C_31_H_48_O_12_ Exact mass: 612.31; Mol. Wt.: 612.71; C, 60.77; H, 7.90; O, 31.34 IUPAC: NA Part used: AeP Extracts: AW^†^ Pharmacology: antimicrobial [134]	**Echiodinin** Chemical properties: C_16_H_12_O_5_ Mol. Wt.: 285 IUPAC: NA Part used: *in vitro *Callus^¶^ Extracts: M^¶^, A^¶^ Pharmacology: antibacterial, antioxidant [141]

Flavonoids		

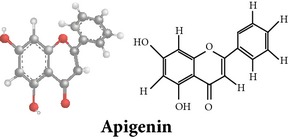	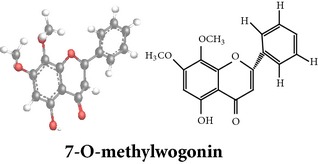	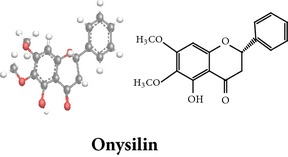
Chemical properties: C_15_H_10_O_4_ Exact mass: 254.06; Mol. Wt.: 254.24; C, 70.86; H, 3.96; O, 25.17 IUPAC: 5,7-dihydroxy-2-phenyl-4H-chromen-4-one Part used: WP^ø^ Extracts: M^ø^ Pharmacology: antiplatelet aggregator [72]	Chemical properties: C_17_H_14_O_5_ Exact mass: 298.08; Mol. Wt.: 298.29; C, 68.45; H, 4.73; O, 26.82 IUPAC: 5-hydroxy-7,8-dimethoxy-2-phenyl-4H- chromen-4-one Part used: WP^ø^ Extracts: M^ø,*£*^, H^¥^ Pharmacology: antiplatelet aggregator [72]	Chemical properties: C_17_H_16_O_5_ Exact mass: 300.1; Mol. Wt.: 300.31; C, 67.99; H, 5.37; O, 26.64 IUPAC: (S)-2,3-dihydro-5-hydroxy-6,7-dimethoxy-2-phenylchromen-4-one Part used: WP^ø^ Extracts: M^ø^ Pharmacology: antiplatelet aggregator [72]

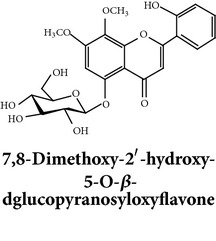	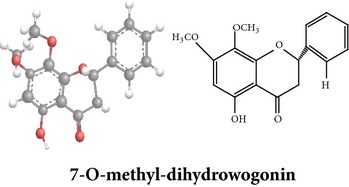	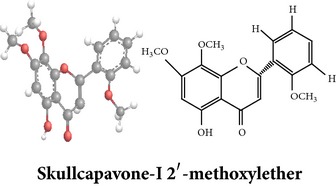
Chemical properties: C_23_H_25_O_11_ Exact mass: 477.1391; Mol. Wt.: 477.1391 IUPAC: NA Part used: AeP^~^ Extracts: E^~^ Pharmacology: antiproliferative [142]	Chemical properties: C_17_H_16_O_5_ Exact mass: 300.1; Mol. Wt.: 300.31; C, 67.99; H, 5.37; O, 26.64 IUPAC: (S)-2,3-dihydro-5-hydroxy-7,8-dimethoxy-2-phenylchromen-4-one Part used: AeP^~^ Extracts: E^~^ Pharmacology: antiproliferative [142]	Chemical properties: C_18_H_16_O_6_ Exact mass: 328.09; Mol. Wt.: 328.32; C, 65.85; H, 4.91; O, 29.24 IUPAC: 5-hydroxy-7,8-dimethoxy-2-(2-methoxyphenyl)-4H- chromen-4-one Part used: AeP^~^ Extracts: E^~^ Pharmacology: antiproliferative [142]

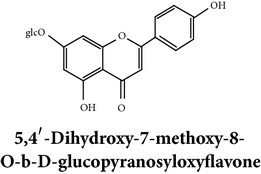	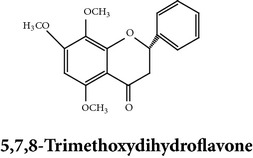	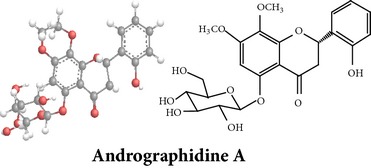
Chemical properties: NA IUPAC: NA Part used: AeP^~^ Extracts: E^~^ Pharmacology: antiproliferative [142]	Chemical properties: NA IUPAC: NA Part used: AeP^~^ Extracts: E^~^ Pharmacology: antiproliferative [142]	Chemical properties: C_23_H_26_O_10_ Exact mass: 462.152597 Mol. Wt.: 462.44654 IUPAC: (2S)-7,8-dimethoxy-2-phenyl-5-[(2S,3R,4S,5S,6R)-3,4,5-trihydroxy-6-(hydroxymethyl)oxan-2-yl]oxy-2,3-dihydrochromen-4-one Part used: AeP^~^ Extracts: E^~^ Pharmacology: antiproliferative [142]

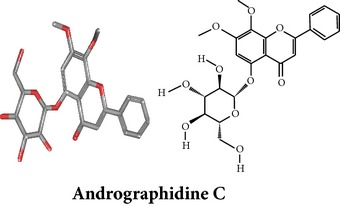	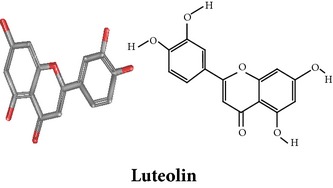	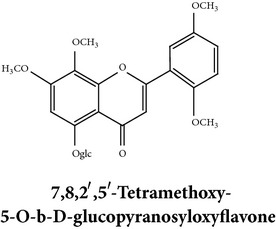
Chemical properties: C_23_H_24_O_10_ Mol. Wt.: 460.43066, EM: 460.136947 IUPAC: 7,8-dimethoxy-2-phenyl-5-[(2S,3R,4S,5S,6R)-3,4,5-trihydroxy-6-(hydroxymethyl)oxan-2-yl]oxychromen-4-one Part used: AeP^~^ Extracts: E^~^ Pharmacology: antiproliferative [142]	Chemical properties: C_15_H_10_O_6_ Exact mass: 286.047738, Mol. Wt.: 286.2363, IUPAC: 2-(3,4-dihydroxyphenyl)-5,7-dihydroxychromen-4-one Part used: AeP^~^ Extracts: E^~^ Pharmacology: antiproliferative [142]	Chemical properties: — IUPAC: — Part used: AeP^~^ Extracts: E^~^ Pharmacology: antiproliferative [142]

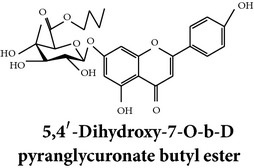	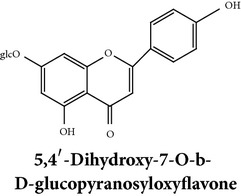	
Chemical properties: — IUPAC: — Part used: AeP^~^ Extracts: E^~^ Pharmacology: antiproliferative [142]	Chemical properties: — IUPAC: — Part used: AeP^~^ Extracts: E^~^ Pharmacology: antiproliferative [142]	

Xanthones		

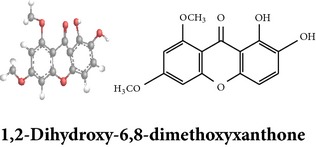	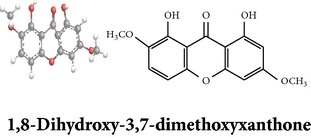	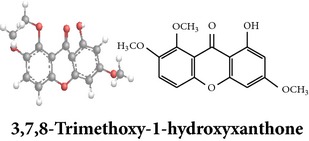
Chemical properties: C_15_H_12_O_6_ Exact mass: 288.06; Mol. Wt.: 288.25; C, 62.50; H, 4.20; O, 33.30 IUPAC: 1,2-dihydroxy-6,8-dimethoxy-9H-xanthen-9-one Part used: R Extracts: sequential extraction with PtE, M, CHCl_3_ and water Pharmacology: antimalarial [143]	Chemical properties: C_15_H_12_O_6_ Exact mass: 288.06; Mol. Wt.: 288.25; C, 62.50; H, 4.20; O, 33.30 IUPAC: 1,8-dihydroxy-2,6-dimethoxy-9H-xanthen-9-one Part used: R Extracts: sequential extraction with PtE, M, CHCl_3_ and water Pharmacology: antimalarial [143]	Chemical properties: C_16_H_14_O_6_ Exact mass: 302.08; Mol. Wt.: 302.28; C, 63.57; H, 4.67; O, 31.76 IUPAC: 8-hydroxy-1,2,6-trimethoxy-9H-xanthen-9-one Part used: R Extracts: sequential extraction with PtE, M, CHCl_3_ and water Pharmacology: antimalarial [143]

Xanthones	Quinic acids	

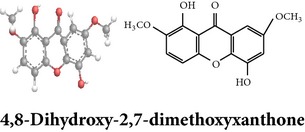	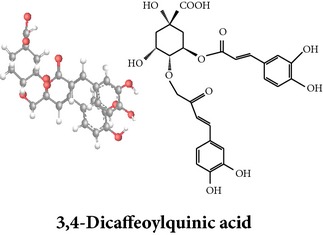	
Chemical properties: C_15_H_12_O_6_ Exact Mass: 288.06; Mol. Wt.: 288.25; C, 62.50; H, 4.20; O, 33.30 IUPAC: 1,5-dihydroxy-2,7-dimethoxy-9H-xanthen-9-one Part used: R Extracts: sequential extraction with PtE, M, CHCl_3_ and water Pharmacology: antimalarial [143]	Chemical properties: C_26_H_26_O_12_ Exact mass: 530.14; Mol. Wt.: 530.48; C, 58.87; H, 4.94; O, 36.19 IUPAC: (1S,3R,4R,5R)-3-((E)-3-(3,4-dihydroxyphenyl)acryloyloxy)-4-((E)-4-(3,4-dihydroxyphenyl)-2-oxobut-3-enyloxy)-1,5-dihydroxycyclohexanecarboxylic acid Part used: WP Extracts: M^ø^ Pharmacology: antiplatelet aggregator [72]	

L: leaves, AeP: aerial parts, WP: whole plants, R: roots, M: methanol, E: ethanol, H: hexane, A: acetone, AW: acetone water, PtE: petroleum ether, CHCl_3_: chloroform, DCM: dichloromethane, Mol. Wt.: molecular weight.

*References*: Arifullah^¶^ et al. 2013 [141]; Chen^*^ et al. 2006 [144]; Chen^#^ et al. 2008 [138]; Chen^~^ et al. 2014 [142]; Fujita^§^ et al. 1984 [145]; Ji^β^ et al. 2005 [135]; Liu^æ^ et al. 2008 [136]; Matsuda^¤^ et al. 1994 [18]; Pramanick^¢^ et al. 2006 [146]; Rao^£^ et al. 2004 [147]; Reddy^¥^ et al. 2003 [148]; Reddy^œ^ et al. 2005 [80]; Seth^‡^ et al. 2010 [149]; Shen^†^ et al. 2006 [134]; Sule^©^ et al. 2011 [121]; Sule^®^ et al. 2012 [123]; Wang^¬^ et al. 2009 [139]; Wu^ø^ et al. 2008 [72]; Xu^$^ et al. 2010 [150]; Zhou^þ^ et al. 2008 [151]; Zou^ð^ et al. 2010 [152].
